# Prevalence and Associated Factors of Sarcopenic Obesity Among Nursing Home Residents: A Cross‐Sectional Multi‐Centre Study

**DOI:** 10.1002/jcsm.13821

**Published:** 2025-05-08

**Authors:** Doris Eglseer, Hristo Hristov, Sanja Krušič, Nadan Gregorič, Irena Hren, Igor Pravst, Živa Lavriša

**Affiliations:** ^1^ Medical University of Graz Graz Austria; ^2^ Institute of Nutrition Ljubljana Slovenia; ^3^ National Institute of Public Health Ljubljana Slovenia; ^4^ University Medical Centre Ljubljana Ljubljana Slovenia; ^5^ Faculty of Medicine University of Ljubljana Ljubljana Slovenia; ^6^ General Hospital of Novo Mesto Novo Mesto Slovenia; ^7^ VIST ‐ Faculty of Applied Sciences Ljubljana Slovenia; ^8^ Biotechnical Faculty University of Ljubljana Ljubljana Slovenia

**Keywords:** nursing home, obesity, older adult, sarcopenia, sarcopenic obesity

## Abstract

**Background:**

Obesity and sarcopenia are prevalent among older adults and associated with adverse health outcomes. The aims of the present study were to assess the prevalence of sarcopenic obesity, to evaluate the co‐occurence of sarcopenia, obesity and malnutrition (risk) and to assess the association between specific characteristics and sarcopenic obesity/probable sarcopenic obesity in nursing home residents.

**Methods:**

Three hundred eighty‐seven nursing home residents with low to moderate care requirements from 20 nursing homes in Slovenia participated in the cross‐sectional NutriCare study. Data on general patient characteristics, physical activity, usual dietary intake (estimated by a 2 × 24 h dietary recall and a food frequency questionnaire), malnutrition (risk) status (Mini Nutritional Assessment [MNA]), laboratory parameters, hand grip strength and body composition (estimated via BIA) were collected. Obesity was defined as a high body fat percentage. Sarcopenia was defined according to the European Working Group on Sarcopenia in Older People2 (EWGSOP2) criteria. Descriptive statistics were used to characterise the sample. Uni‐ and multivariable binary logistic regression analyses were performed to explore associations between the predictor variables and sarcopenic obesity.

**Results:**

The prevalence of obesity was 90.7% according to high fat mass and 38.3% according to BMI (≥ 30). Prevalences were 27.6% (sarcopenia) and 24.5% (sarcopenic obesity), respectively. Probable sarcopenic obesity (low hand grip strength combined with obesity) was found in 37.6% of participants. A co‐occurence of malnutrition (risk) and sarcopenia was present in 11.9%, whereas a combination of malnutrition (risk) and obesity was found in 28.2%. In 9.6% of the participants, a combination of all three phenomena—sarcopenia, obesity and malnutrition (risk)—was identified. The multivariable logistic regression model shows that higher age (OR 1.07; CI 1.02, 1.11), male sex (OR 2.3; CI 1.22, 4.5) and higher energy intake (OR 1.13; CI 1.04, 1.22) were significantly associated with sarcopenic obesity. Male sex (OR 2.30; CI 1.33, 3.98), higher age (OR 1.07; CI 1.03, 1.11), higher care requirements (OR 2.14; CI 1.20, 3.79), lower MNA score (OR 0.88; CI 0.80, 0.97) and metabolic equivalent of tasks (MET minutes/week) (OR 0.99; CI 0.98, 1.00) were significantly associated with probable sarcopenic obesity.

**Conclusions:**

The study indicates a notable prevalence of obesity and sarcopenic obesity among nursing home residents with lower to moderate care dependency. These findings underscore the importance of optimising nutritional intake and other modifiable lifestyle factors associated with such health conditions and implementing them into targeted, individualised interventions to reduce the risk of obesity and sarcopenic obesity and to improve health outcomes.

## 1 Introduction

Obesity and sarcopenia are prevalent among older adults. Approximately 20% are affected by obesity [1] and up to 16% by sarcopenia [2], depending on the used criteria. The prevalence of sarcopenia can be up to 66% in patients with certain diagnoses, which is much higher than in the general older population [2]. As individuals age, an increasing number experience both conditions simultaneously, a state known as sarcopenic obesity (SO) [3]. Sarcopenia and obesity, as well as the combination of the two, are associated with several negative health outcomes in older adults, including functional decline, disability, hospitalisation, metabolic impairments such as insulin resistance, and other comorbidities [4, 5].

A high prevalence of sarcopenia and obesity can be observed in nursing homes. Obesity is present in around 17% of European nursing home residents [6] and sarcopenia in around 60% [7], respectively. Only limited prevalence data are available for SO in nursing homes, and these data vary significantly across current studies, ranging from 0% in a small cohort of German nursing home residents to 45% in a larger cohort of Chinese residents [8, 9, 10]. The various prevalence rates depend heavily on the definitions used for obesity and sarcopenia. However, due to the sedentary lifestyle of older adults, a significant risk factor for sarcopenia [5], experts expect these prevalence rates to also rise in nursing home residents during the next decades.

Furthermore, being admitted to a nursing home seems to increase the risk of developing sarcopenia. In newly admitted nursing home residents, around one in four obese residents experiences substantial weight loss during their first year in the nursing home [11]. This is a potential health threat for these residents because studies show that weight loss in older age is accompanied by a substantial loss of lean mass [12]. Moreover, obese residents who have experienced unintentional weight loss also suffer from increased functional and cognitive impairments [11]. In this specific group of nursing home residents, the ‘obesity paradox,’ which refers to the observation that overweight and even mildly obese older adults have better survival rates than those who are of normal weight or underweight, seems to not apply [13]. If obesity is combined with muscle weakness, as seen in sarcopenia, this seemingly protective effect is no longer present [14].

Another challenge in nursing home residents is the high risk of malnutrition. Up to half of nursing home residents are at risk of malnutrition [15], and malnutrition develops in one in 10 indviduals within 6 months of being admitted to a nursing home [16]. Malnutrition can also occur in overweight or obese individuals and is often masked by the overweight of the affected persons [17].

In general, existing epidemiological data on SO in nursing home residents are limited and heterogenous. There is also a lack of detailed understanding regarding how certain characteristics are associated with the prevalence of sarcopenia and SO in this specific population. The aims of this study were (1) to assess the prevalence of SO in nursing home residents, (2) to evaluate the co‐occurrence of sarcopenia, obesity and malnutrition in nursing home residents and (3) to assess the association of specific characteristics with SO/probable SO in nursing home residents.

## 2 Material and Methods

### 2.1 Study Design and Recruitment of Participants

This study was conducted as part of the Slovenian cross‐sectional multi‐centre NutriCare study, which investigated the nutritional status of and dietary challenges faced by nursing home residents in all nine Slovenian health regions. The study protocol, inclusion/exclusion criteria and methods have been previously described in detail elsewhere [18]. A total of 387 subjects with low to moderate care requirement levels and without severe cognitive impairment (according to nursing home records) participated in the study. Residents' care requirement categories are determined by nursing homes according to the legislation on social care. Low to moderate care categories were 1–3a [19].

### 2.2 Data Collection

General information and data on the participants' medical histories, diagnoses and prescribed medications were collected by screening their nursing home records. A general questionnaire was administered during interviews led by trained researchers to assess sociodemographic factors, health statuses, eating habits, food allergies and supplement usage as previously described [18].

### 2.3 Dietary Intake

The participants' dietary habits were assessed according to the European Food Safety Authority (EFSA) guidelines by conducting two 24‐h dietary recall (24HDR) interviews on 2 days at least 7 days apart. To model the usual intakes, the 24HDR were complemented by a food frequency questionnaire (FFQ) which had been used to collect the frequency of consumption within specific food categories over the previous 12 months. Nutrient intakes were estimated using the Open Platform for Clinical Nutrition online dietary assessment tool, which includes a database of nutrient contents for specific foods typically consumed in Slovenia [20]. The Multiple Source Method (MSM) was employed to model variations in intake distribution, considering the variability in food consumption over time and among different individuals [21].

### 2.4 Malnutrition

The malnutrition risk category was assessed using the long form of the Mini Nutritional Assessment (MNA) questionnaire [22]. Participants were divided into three groups based on their MNA scores reflecting their risk of malnutrition: < 17 (malnourished), 17–23.5 (at risk of malnutrition) and 24–30 (satisfactory nutritional status).

### 2.5 Anthropometric and Body Composition Data

Participants' height and weight were assessed using the Seca 799 medical scale (Seca GmbH, Hamburg, Germany). A Bodystat Multiscan 5000 (Bodystat, Isle of Man, Ireland) multifrequency bioelectrical impedance monitor (frequency range 5–500 kHz) was used to estimate participants' body composition. Measurements were performed on all individuals without cardiac pacemakers who were able to assume the required measurement position (*N* = 332). Fat mass and fat‐free mass were estimated using the equation of Deurenberg et al., which was proposed for use in populations above 60 years of age [23]. Appendicular lean soft tissue mass (ALST) was calculated for each participant based on their raw bioelectrical impedance data (reactance and resistance) and anthropometric data using the equation developed by Sergi et al. [24], which provides a valid estimation of ALST in older Caucasian adults.

SO was defined as the simultaneous presence of sarcopenia and obesity. The assessment of sarcopenia was performed on the basis of the EWGSOP2 criteria [25]. Probable sarcopenia was defined by low hand grip strength (< 27 kg in men and < 16 kg in women). Sarcopenia was defined as both low hand grip strength and low ALST (< 20 kg in men and < 15 kg in women). Obesity was defined based on fat mass percentage (> 30% for males and > 42% for females). All cutoff points used for the assessment of sarcopenia and obesity can be found in Table 1.

**TABLE 1:** Sex‐specific cutoff points used for the assessment of sarcopenia and obesity.

	Cutoff points	
	Males	Females
Hand grip strength [24]	< 27 kg	< 16 kg
Appendicular skeletal muscle mass (ASMM) [24]	< 20 kg	< 15 kg
Timed Up and Go test [24]	≥ 20 s	≥ 20 s
Body mass index	> 30 kg/m^2^	> 30 kg/m^2^
Body fat (%) [25]	> 30%	> 42%
Waist/hip ratio [26]	0.9	0.85

### 2.6 Physical Activity (PA)

The participants' PA was evaluated using the short form of the International Physical Activity Questionnaire (IPAQ), and MET scores were calculated using the established methodology [26]. Total PA of less than 600 MET‐minutes/week was considered a low IPAQ score; at least 600 MET‐minutes/week was considered a moderate IPAQ score; and at least 3000 MET‐minutes/week was considered a high IPAQ score. Additionally, the typical PA throughout adulthood was self‐assessed as part of the general questionnaire (low, medium or moderate).

### 2.7 Hand Grip Strength

Hand grip strength was measured by using a Jamar hydraulic hand dynamometer (J. A. Preston Corporation, Clifton, NJ). Seated participants were positioned with their shoulders adducted and neutrally rotated, elbows flexed at a 90° angle, forearms in a neutral position and wrists positioned between 0° and 30° of dorsiflexion. The participants performed the measurement using their dominant hand and repeated it three times, pausing between measurements (about 15 s). The highest grip score from the three measurements was used for analysis.

### 2.8 Timed Up and Go Test

The Timed Up and Go test (TUG) is a reliable and valid test used to quantify functional mobility in older adults [27]. In this study, participants were asked to sit comfortably on a standard chair, with their back against the back of the chair and their arms resting on the chair arms. Upon hearing the word ‘go’, they were to stand up, walk to a marker 3 m away in a safe pace, turn around, walk back and sit down again. The time needed to accomplish this task was recorded. The task was repeated three times, with a pause between each cycle. The average of the three measured times was used for data analyses.

### 2.9 Laboratory Blood Biomarker Analysis

A fasting venous blood sample was collected from each of the participants and transported to an accredited medical diagnostic laboratory (Adrialab/Synlab, Ljubljana, Slovenia) for same‐day analysis. For the present study, we only used data for the C‐reactive protein (CRP) concentration as a marker of inflammation, with a cutoff value of 5 mg/L being used.

### 2.10 Data Analyses

First, all variables of interest were analysed descriptively. Dichotomous variables are given as percentages and absolute numbers. Metric data are given as means and standard deviations (SDs) or median and interquartile ranges (IQRs), as deemed appropriate. A binary logistic regression analysis using the enter method was conducted to determine which specific characteristics are related to (probable) SO in nursing home residents, establishing probable SO and SO as the dependent variables. Independent variables were chosen based on the available literature: sex, age, BMI, current smoking, care requirement levels, diagnoses of chronic diseases, inflammation (CRP), vitamin D supplementation, malnutrition according to the MNA, energy intake and protein intake. Variables with *p* > 0.2 in the univariable regression analysis were included in the multiple logistic regression model, where sex and age were defined as additional important factors of interest, even if these failed to reach *p* > 0.2 in the univariable analysis. The variance inflation factor (VIF) was analysed prior to the regression analysis to avoid multicollinearity of the independent variables, and a VIF < 4 was considered acceptable. Goodness‐of‐fit was assessed by carrying out the Hosmer–Lemeshow test and the Omnibus Test of Model Coefficients. To test the significance and variance of the model, we calculated Cox and Snell's *R* ^2^ and Nagelkerke's *R* ^2^. We used IBM SPSS Version 27 (IBM SPSS, IBM Corp., Armonk, NY) to perform all statistical analyses.

## 3 Results

**FIGURE 1:**
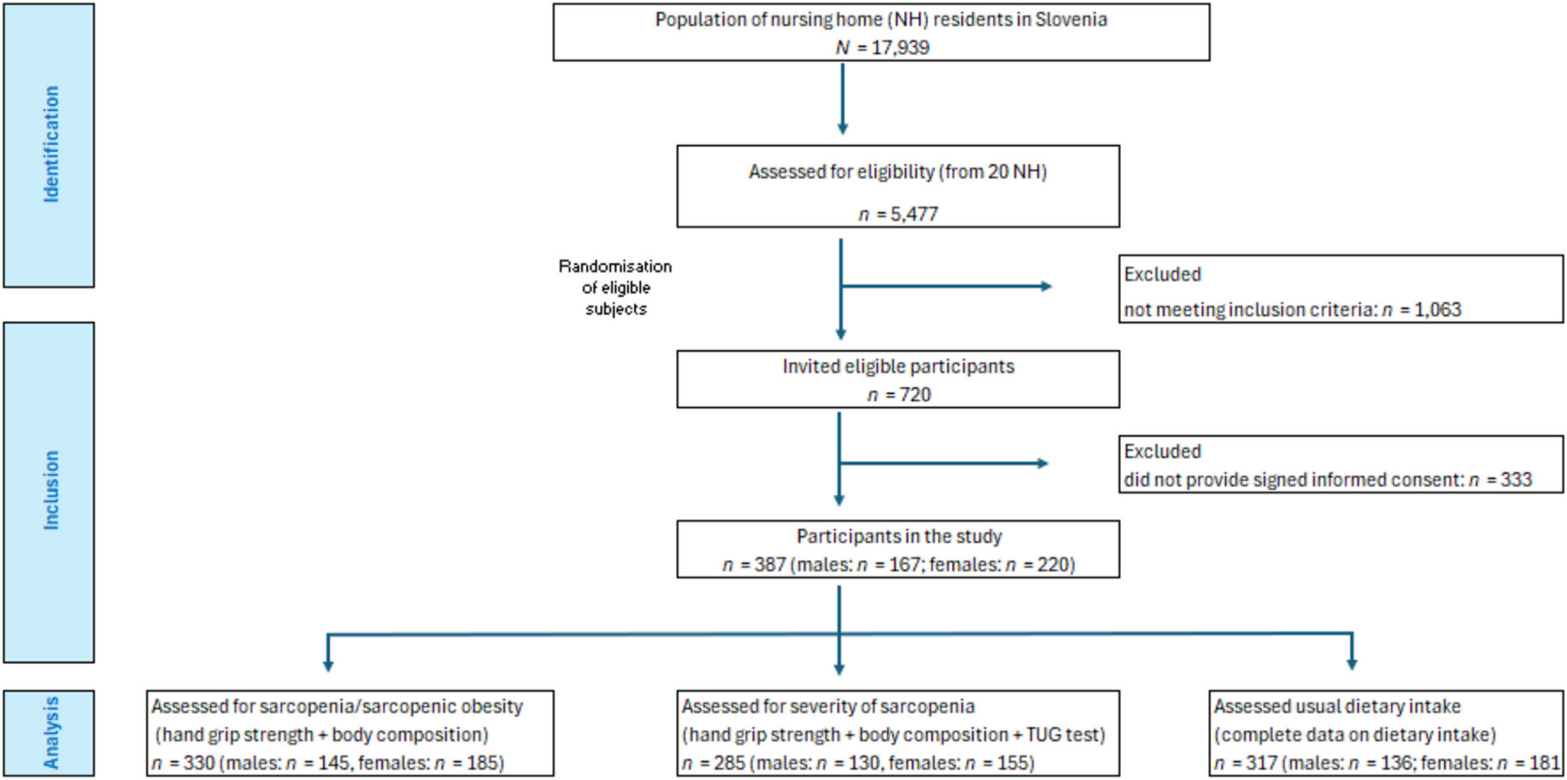
Participant flow chart.

In total, 387 nursing home residents participated in the study. The recruitment of participants is shown in Figure 1. More than half (56.8%) of these were female and had a mean age of 81.5 years (range 65–101). The majority (73.1%) of them had low care requirement levels. We were not able to collect complete measurements for all participants; therefore, data on hand grip strength is missing for four participants, data on body composition (BIA) for 55 participants and data on the TUG for 72 participants, respectively. We did not calculate BMI for one participant because it was not possible to obtain a reliable height measurement with our equipment due to the participant's specific body characteristics.

The prevalence of sarcopenia was 27.6% (*n* = 91), while the prevalence of probable sarcopenia (low hand grip strength with normal ALST) was 14.2% (*n* = 47). The prevalence of obesity according to high fat mass was 90.7% (*n* = 301) and 38.3% according to BMI ≥ 30 (*n* = 148), respectively. The participants' characteristics are displayed in Table 2.

**TABLE 2:** Characteristics of study subjects divided according to the diagnosis of obesity, probable sarcopenic obesity and/or sarcopenic obesity.

	Total sample (*n* = 387)	Obesity (*n* = 301)	Probable sarcopenic obesity (*n* = 124)	Sarcopenic obesity^b^ (*n* = 81)
Female sex, % (*n*)	56.8 (220)	53.8 (162)	50.0 (62)	54.3 (44)
Age, mean (± SD)	81.5 (± 7.8)	80.9 (± 7.9)	83.3 (± 7.9)	84.4 (± 7.7)
BMI, mean (± SD)^a^	28.8 (± 5.7)	29.6 (± 5.5)	28.4 (± 5.2)	26.9 (± 4.3)
Waist‐to‐hip ratio, mean (± SD)	0.95 (± 0.1)	0.96 (± 0.09)	0.9 (± 0.2)	0.9 (± 0.2)
Current smoker, % (*n*)^a^	14.3 (54)	16.0 (47)	13.6 (16)	14.7 (11)
Number of chronic diseases, median (IQR)	2 (1–3)	2 (1–3)	2 (1–3)	2 (1–3)
CRP > 5, % (*n*)	40.4 (155)	40.9 (123)	43.1 (53)	38.3 (31)
Vitamin D supplementation, % (*n*)	56.1 (217)	55.8 (168)	54.0 (67)	53.1 (43)
TUG test result ≥ 20 s, % (*n*)	21.9 (69)	19.4 (50)	27.5 (28)	31.8 (21)
Frequent diagnoses % (*n*)				
Diabetes mellitus type 2	23.0 (89)	22.0 (66)	24.2 (30)	17.3 (14)
Kidney disease	14.2 (55)	13.0 (39)	16.1 (20)	9.9 (8)
Osteoporosis	12.4 (48)	10.7 (32)	15.3 (19)	22.2 (18)
Heart failure	10.6 (41)	9.3 (28)	11.3 (14)	8.6 (7)
Care dependency, % (*n*)^a^				
Low care requirements	70.3 (272)	73.1 (220)	59.7 (74)	60.5 (49)
Medium care requirements	25.8 (100)	24.3 (73)	35.5 (44)	33.3 (27)
High care requirements	3.9 (15)	2.7 (8)	4.8 (6)	6.2 (5)
Malnutrition risk (MNA)^a^				
Normal nutritional status	60.6 (234)	63.8 (192)	53.2 (66)	54.3 (44)
Risk of malnutrition	36.8 (142)	35.0 (105)	44.4 (55)	43.2 (35)
Malnutrition	2.6 (10)	1.3 (4)	2.4 (3)	2.5 (2)

^a^ Mini nutritional assessment (MNA): missing data for 1 resident; C‐reactive protein (CRP): missing data for 4 residents (3 obese, 1 probable sarcopenic obesity (SO), 1 SO); smoker: missing data for 9 residents (7 obese, 6 probable SO and 6 SO); Timed Up and Go (TUG) test: missing data for 72 residents (42 obese, 22 probable SO, 15 SO); and body mass index (BMI) implausible data for 1 resident (total sample).

^b^ Sarcopenic obesity refers to low hand grip strength and low ALST combined with high fat mass. Probable sarcopenic obesity refers to low hand grip strength combined with high fat mass.

### 3.1 Prevalence of SO

Probable SO (low hand grip strength combined with obesity) was present in 37.6% (*n* = 124) of the participants. The prevalence of SO (sarcopenia and obesity) was 24.5% (*n* = 81), with 23.8% (*n* = 44) being observed in females and 25.5% (*n* = 37) in males.

### 3.2 Co‐Occurence of Malnutrition, Sarcopenia and Obesity

**FIGURE 2:**
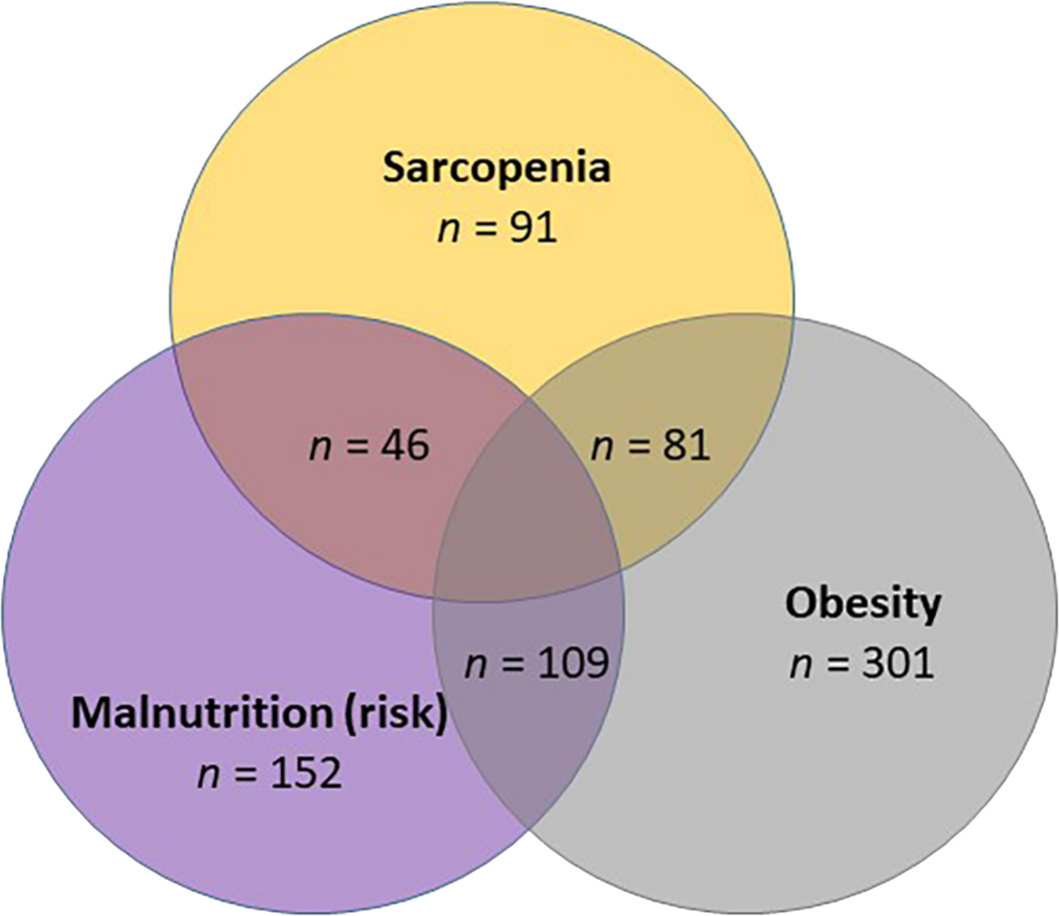
Overlap between sarcopenia, obesity and malnutrition in the Nutricare study participants.

Malnutrition (risk) and sarcopenia co‐occured in 11.9% (*n* = 46) of the participants, whereas a combination of malnutrition (risk) and obesity was present in 28.2% (*n* = 109). A combination of all three conditions—sarcopenia, obesity and malnutrition (risk)—was identified in 9.6% (*n* = 37) of the participants (see Figure 2).

### 3.3 Association Between Specific Characteristics and SO

Univariable analyses show an association between SO and higher age, higher care requirement levels, higher energy and protein intake, and an osteoporosis diagnosis. The final multivariable logistic regression model revealed an association between SO and higher age (OR 1.07; CI 1.02, 1.11), male sex (OR 2.3; CI 1.22–4.5) and higher energy intake (OR 1.13; CI 1.04, 1.22).

When using probable SO as the dependent variable in the univariable analysis, an association was identified between the probable SO and higher age, higher care requirement levels, MET minutes/week and a lower MNA score. The final multivariable logistic regression model yielded an association between probable SO with male sex (OR 2.30; CI 1.33, 3.98), higher age (OR 1.07; 1.03, 1.11), higher care requirement levels (OR 2.14; CI 1.20, 3.79), a lower MNA score (OR 0.88; IC 0.80, 0.97) and decreased MET/minutes/week (OR 0.99; CI 0.98, 1.00). The results of the univariable and multiple logistic regression analyses are displayed in Table 3.

**TABLE 3:** Results of the univariable and multiple logistic regression analyses with sarcopenic obesity and probable sarcopenic obesity^b^ as the dependent variables.

	Univariable logistic regression analysis						Multivariable logistic regression analysis^a^					
Independent variables	B	SE	*p*	OR	95% CI		B	SE	*p*	OR	95% CI	
Dependent variable: sarcopenic obesity												
Male sex (reference: females)	0.09	0.26	0.727	1.10	0.66	1.82	0.85	0.33	0.010	2.34	1.22	4.47
Age	0.08	0.01	< 0.001	1.08	1.04	1.12	0.07	0.02	0.002	1.07	1.02	1.11
Care category												
Medium care requirements (reference: low care requirements)	0.68	0.29	0.017	1.98	1.13	3.48	0.46	0.33	0.166	1.59	0.83	3.04
Smoking	−0.29	0.30	0.326	0.75	0.42	1.34						
Former smoker	−0.26				0.36							
Smoker (reference: non‐smoker)		0.38	0.502	0.77		1.64						
Number of chronic diseases	0.06	0.10	0.523	1.06	0.88	1.28						
IPAQ score												
Moderate IPAQ score	−0.07	0.28	0.798	0.93	0.54	1.60						
High IPAQ score (reference: low IPAQ score)	−0.11	0.61	0.864	0.90	0.27	3.00						
MET (minutes/week)	0.001	0.001	0.130	1.00	0.99	1.01	0.001	0.00	0.058	1.000	0.99	1.01
CRP concentration	0.01	0.01	0.446	1.01	0.99	1.02						
Vitamin D supplement use	−0.24	0.25	0.347	0.79	0.47	1.30						
Physical activity in adulthood	0.11	0.21	0.606	1.11	0.74	1.68						
MNA score	−0.07	0.04	0.108	0.93	0.86	1.02	−0.08	0.06	0.167	0.93	0.83	1.03
Energy intake, kcal/kg/day	0.09	0.02	< 0.001	1.10	1.06	1.14	0.12	0.04	0.003	1.13	1.04	1.22
Protein intake, g/kg/day	1.65	0.43	< 0.001	5.22	2.26	12.09	−0.31	0.93	0.736	0.73	0.12	4.50
Diagnosed diabetes mellitus type 2	−0.40	0.33	0.229	0.67	0.35	1.28						
Diagnosed heart failure	−0.12	0.45	0.790	0.89	0.37	2.14						
Diagnosed kidney disease	−0.50	0.41	0.228	0.61	0.27	1.36						
Diagnosed osteoporosis	1.08	0.35	0.002	2.95	1.49	5.83	0.77	0.43	0.076	2.16	0.92	5.03
Dependent variable: probable sarcopenic obesity												
Male sex (reference: females)	0.39	0.23	0.086	1.48	0.95	2.32	0.83	0.28	0.003	2.30	1.33	3.98
Age	0.06	0.02	< 0.001	1.07	1.03	1.10	0.07	0.02	< 0.001	1.07	1.03	1.11
Care category												
Medium care requirements (reference: low care requirements)	1.00	0.27	< 0.001	2.73	1.62	4.58	0.76	0.29	0.009	2.14	1.20	3.79
Smoking												
Former smoker	−0.23	0.26	0.380	0.78	0.48	1.32						
Smoker (reference: non‐smoker)	−0.40	0.34	0.240	0.67	0.34	1.31						
Number of chronic diseases	0.16	0.09	0.066	1.17	0.99	1.39	0.03	0.10	0.747	1.03	0.85	1.27
IPAQ score												
Moderate IPAQ score	−0.36	0.24	0.137	0.70	0.43	1.12	0.72	0.40	0.067	2.06	0.95	4.49
High IPAQ score (reference: low IPAQ score)	−0.34	0.54	0.531	0.71	0.25	2.07						
MET minutes/week	0.001	0.001	0.019	1.00	0.99	1.01	−0.001	0.00	0.028	0.99	0.99	1.00
CRP	0.001	0.01	0.814	1.00	0.99	1.02						
Vit. D supplementation	−0.23	0.23	0.313	0.79	0.51	1.24						
Physical activity in adulthood	0.19	0.18	0.310	1.21	0.84	1.73						
MNA score	−0.12	0.04	0.003	0.89	0.82	0.96	−0.13	0.05	0.008	0.88	0.80	0.97
Energy intake, kcal/kg/day	0.03	0.01	0.080	1.03	1.00	1.06	0.04	0.02	0.051	1.04	1.00	1.08
Protein intake, g/kg/day	0.44	0.37	0.234	1.56	0.75	3.22						
Diagnosed diabetes mellitus type 2	0.19	0.27	0.482	1.21	0.71	2.06						
Diagnosed heart failure	0.35	0.38	0.361	1.42	0.67	2.98						
Diagnosed kidney disease	0.29	0.32	0.374	1.33	0.71	2.50						
Diagnosed osteoporosis	0.47	0.34	0.169	1.59	0.82	3.10	0.22	0.41	0.590	1.25	0.56	2.79

Abbreviations: CRP = C‐reactive protein, IPAQ = International Physical Activity Questionnaire, MET = metabolic equivalent of task, MNA = Mini Nutritional Assessment.

^a^ Variables were included in the multivariable logistic regression model if they reached at least *p* > 0.2 in the univariable analysis. Continuous variables: age, number of chronic diseases, metabolic equivalent (MET) minutes/week, C‐reactive protein (CRP), Mini Nutritional Assessment (MNA) score, energy intake and protein intake.

^b^ Sarcopenic obesity refers to low hand grip strength and low ASMM combined with high fat mass. Probable sarcopenic obesity refers to low hand grip strength combined with high fat mass.

For both final multivariable logistic regression models, the Omnibus Test of Model Coefficients yielded *p*‐values < 0.001, indicating the significant predictive value of the final regression models. The goodness‐of‐fit of the models was assessed by applying the Hosmer–Lemeshow test, which shows a *p*‐value > 0.05, confirming good model fit. Additionally, Nagelkerke's *R* ^2^ indicated an acceptable amount of explained variance [28].

## 4 Discussion

This study was carried out to assess the prevalence and associated factors of SO in nursing home residents. The prevalence of SO in this study population was 24.5%, while probable SO (measured by low hand grip strength combined with obesity) was present in 37.6% of the participants. In the final multivariable logistic regression model, the factors of higher age, male sex and higher energy intake were significantly associated with the presence of SO. The factors of male sex, higher age, higher care requirement levels, a lower MNA score and MET (minutes/week) were significantly associated with probable SO.

We found a relatively high prevalence of SO in this study. This may be explained by the high prevalence of obesity in this cohort (90.7%), which was defined as a fat mass percentage > 42% for females and > 30% for males. Fat mass was estimated by conducting bioimpedance measurements and applying the equation of Deurenberg et al., which was developed and validated specifically for European older individuals [23]. This equation is recommended as a default for geriatric populations by the producers of the Bodystat Multiscan 5000 device and is widely used in clinical practice [23]. Most validation studies using the currently available BIA equations were conducted on generally healthy older individuals which might differ from nursing home populations. Currently, no specific BIA equations are available that are tailored to nursing home residents, and the development of such equations could be a valuable subject of future research. This explains why the use of different methods to estimate fat mass by means of bioimpedance data in such population groups may yield somewhat varying results [29]. However, in the absence of direct measurements by means of dual‐energy X‐ray absorptiometry (DEXA) or magnetic resonance imaging (MRI) and computed tomography (CT) (which was not applicable for our study), we found that the approach used in this study provided the most precise estimates.

The prevalence of SO depends heavily on the measurements, equations, definitions and cutoff values used for sarcopenia and obesity. Research on SO and debates regarding which cutoff values and measurement methods are suitable for defining SO are ongoing [3]. However, there are no specific recommendations available for older nursing home residents, and data and evidence that indicate how to optimally assess and define SO in this population are largely missing. In general, the data on the prevalence of SO in nursing homes are extremely limited. One German study conducted with 69 nursing home residents, for example, did not identify a single person with SO [9]. These researchers also used the EWGSOP2 criteria to identify sarcopenia [25], as we did in the present study, and BMI and fat mass to define obesity, but the publication does not clearly state which equation was used to calculate total body fat mass. Even if the definition of SO used by these researchers was similar to the one used in this paper, the former population was older and had higher care requirement levels, which might explain why the results deviate. In another study with a population of 397 Turkish nursing home residents, an SO prevalence of 13.3% was detected [8]. However, these authors defined low hand grip strength in combination with obesity (defined as a BMI ≥ 30 kg/m^2^) as indicative of SO. These results are more similar to the prevalence of probable SO in our current sample, which is 37%. In contrast, another European study diagnosed osteosarcopenic obesity in 70.8% of female nursing home residents and in 47.8% of male residents [30]. A Chinese study with 832 participants also revealed a high prevalence of SO, namely, 43.5% in nursing home residents, but both these publications do not clearly state which equation was used to calculate total body fat mass. Equations are (also) population‐specific; therefore, an Asian population cannot be compared with a European population. The observed major differences in the prevalence of SO in nursing home residents confirm the challenge presented by the different measurement methods and cutoff values and specifically with regard to the assessment of body composition. The question remains of how to best calculate fat mass and fat‐free mass for the specific population of nursing home residents [10], when BIA is used for the body composition assessment.

The present study found that higher age, as well as male sex, was associated with SO. The association with higher age was not surprising because previous studies have shown that sarcopenia and the proportion of fat mass increase with advanced age [4, 25]. Data from the NHANES study, for example, confirm a higher SO prevalence in advanced age [31]. However, the association between SO and male or female sex was not entirely expected. Our results are similar to those of a Spanish study with community‐dwelling older adults, with the prevalence of SO being higher in males (29%) than in females (16.5%) [32]. Another study conducted in Germany with community‐dwelling older individuals indicated a very low prevalence of SO in this setting, but it was nevertheless higher in males than in females [33]. However, the NHANES study revealed an SO prevalence of 33% in females (80 years and older, 48%), while only 12.6% of men were classified as being sarcopenically obese (80 years and older, 27.5%) [31]. The discrepancies between these study findings might again indicate that epidemiological data on the prevalence of SO depend heavily on the used assessment methods and (not) on the use of sex‐specific cutoff values.

We found a correlation between higher care requirement levels and SO. It must be noted that the NutriCare study sample included mostly participants with low to medium care requirements, so it is not possible to draw any conclusions for residents with high care requirement levels. Nevertheless, our observation supports the results of former studies in which SO in older adults was related to greater disability, higher care dependency and restrictions in performing simple physical activities such as ascending and descending stairs [34]. For nursing home residents, a higher degree of disability means that they have higher care requirements; this does affect not only the residents themselves but also the nursing home staff. Due to the extensive amount of assistance these residents need to perform activities of daily living, caring for these residents may be more stressful than caring for residents with lower care requirement levels and also raises the costs of care [35]. However, a collaboration of multidisciplinary team members including physicians, dietitians and physical therapists should be standard practice in nursing homes to address these complex health, nutrition and PA challenges. Unfortunately, this is still not the case in many nursing homes.

This study also highlights a substantial co‐occurrence of obesity and malnutrition (risk). Malnutrition was assessed by means of the MNA, which is currently one of the most commonly used malnutrition screening tools in older populations [36]. The MNA evaluates nutritional status with a variety of parameters including body measurements/indexes (e.g., BMI and calf circumference), dietary intake, health status, lifestyle factors and cognitive function. This tool also enables the user to consider subjective perceptions of health and nutrition. Particularly in environments such as nursing homes, residents often exhibit a variety of characteristics which can all contribute to a lower MNA score, indicating potential malnutrition despite an individual's higher body weight, BMI or total body fat mass. We found that a combination of malnutrition (risk) and obesity was present in 28.2% of the nursing home residents. Similarly, a Polish study found that sarcopenia, obesity and SO were often associated with lower MNA scores in elderly subjects [37]. Obesity (defined by BMI) and malnutrition (risk) were prevalent in a Swedish cohort of nursing home residents as well, with 12.5% of obese participants identified as at risk of malnutrition and another 4.2% as malnourished according to the MNA‐SF [38]. In old and very old people, this seems to be a common observation. However, higher BMI has also been associated with lower mortality, regardless of nutritional status [38]. These findings raise the question of whether the MNA is really a reliable and sensitive tool for detecting specific health risks associated with obesity in older adults [39]. Many aspects regarding the optimal nutritional status of older adults and its assessment tools remain unclear, and further research is necessary. Frailty is another important geriatric syndrome in nursing home residents and a significant predictor of all‐cause mortality in this population [40]. Research indicates a notable co‐occurrence of frailty and malnutrition [41]. However, because frailty was not assessed in our study sample, we are unable to make any conclusions on this matter.

The present study findings also underline some important issues with regard to the assessment of older nursing home populations which should be a topic for future research. Our results indicate that it is of utmost importance to develop BIA equations tailored to the estimation of fat mass and fat‐free mass in the specific group of older nursing home residents, including obese subjects, which are common in such populations. These equations are largely lacking, and the development of such equations would enable body composition parameters to be more reliably estimated on the basis of raw BIA data from obese older adults. This aspect is relevant not only for research but also for clinical practice, where the BIA is widely used to assess body composition and clinical interventions often depend on these data.

The strength of the present study is that the sample included nursing home residents from different nursing homes in various geographic regions of Slovenia, providing a good representation of the overall nursing home population in this country. For the assessment of dietary intake, the MSM was employed to model variations in intake distribution, both between and within days and individuals. The MSM combined data from two 24HDR interviews and the FFQ. This enabled a more accurate estimation of long‐term dietary intake because using data from only two 24HDR might not have accurately represented a participant's usual intake. The bias associated with under−/overreporting and misreporting was reduced because of the interviewers' access to the daily menus and portions which were used to help participants remember what and how much they had eaten. One limitation of the study is that body composition was estimated using the BIA, which requires a further estimation of fat mass and fat‐free mass by applying a specific equation; this equation was validated for older European adults but not specifically for nursing home populations. Obesity was defined as the presence of high fat mass, so the choice of equation influences the resulting parameters and, in turn, also the prevalence of obesity and SO. Ideally, body composition would have been measured directly using DEXA, but this was not feasible in our study. The complete assessment process in our study was carried out in the participants' nursing homes. Transportation to other locations would have been required if the DEXA method had been used, and this would have considerably increased the burden on the aged and fragile subjects. The study sample did not include subjects with the highest care dependency; therefore, our study results cannot be extrapolated to this population group. Both the participants' current PA (IPAQ questionnaire) and PA throughout adulthood were self‐reported. For a more accurate assessment of PA, other methods should be employed, such as the use of wearable fitness trackers, accelerometers or pedometers in order to objectively measure movement and intensity.

## 5 Conclusions

This study reveals a marked prevalence of obesity and SO in nursing home residents with lower care dependency in Slovenia. A complex interplay between malnutrition (risk), sarcopenia and obesity was observed, and a notable number of individuals were observed to be affected by multiple conditions simultaneously. The findings stress the need to optimise nutritional intake and implement targeted and tailored interventions to mitigate the risk of obesity and SO as well as the co‐occurrence of different conditions to improve health outcomes.

## Ethics Statement

Ethical approval was obtained from the Slovenian National Medical Ethics Committee (0120‐531/2021/13). The study was registered on clinicaltrials.gov (NCT05389618). All authors certify that they comply with the ethical guidelines for authorship and publishing in the Journal of Cachexia, Sarcopenia and Muscle.

## Conflicts of Interest

The authors declare no conflicts of interest.
